# Validation of beat by beat fetal heart signals acquired from four-channel fetal phonocardiogram with fetal electrocardiogram in healthy late pregnancy

**DOI:** 10.1038/s41598-018-31898-1

**Published:** 2018-09-11

**Authors:** Ahsan Khandoker, Emad Ibrahim, Sayaka Oshio, Yoshitaka Kimura

**Affiliations:** 1 Healthcare Engineering and Innovation Center, Department of Biomedical Engineering, Khalifa University of Science and Technology, Abu Dhabi, 127788 United Arab Emirates; 2 Department of Electrical Engineering, Eindhoven University of Technology, Eindhoven, The Netherlands; 3 Department of Gynecology and Obstetrics, Tohoku University Hospital, Sendai, 980-8577 Japan; 4 Institute of International Advanced Interdisciplinary Research, Tohoku University School of Medicine, Sendai, 980-8577 Japan

## Abstract

Fetal heart rate monitoring is an essential obstetric procedure, however, false-positive results cause unnecessary obstetric interventions and healthcare cost. In this study, we propose a low cost and non-invasive fetal phonocardiography based signal system to measure the fetal heart sounds and fetal heart rate. Phonocardiogram (PCG) signals contain acoustic information reflecting the contraction and relaxation of the heart. We have developed a four-channel recording device with four separated piezoelectric sensors harnessed by a cloth sheet to record abdominal phonogram signals. A multi-lag covariance matrix based eigenvalue decomposition technique was used to extract fetal and maternal heart sounds as well as maternal breathing movement. In order to validate the fetal heart sounds extracted by PCG signal processing, 10 minutes’ simultaneous recordings of fetal Electrocardiogram (fECG) and abdominal phonogram from 15 pregnant women (27 ± 5-year-old) with fetal gestation ages between 33 and 40 weeks were obtained and processed. Highly significant (p < 0.01) correlation (r = 0.96; N = 270) was found between beat to beat fetal heart rate (FHR_ECG_) from fECG and the same (FHR_PCG_) from fetal PCG signals. Bland–Altman plot of FHR_ECG_ and FHR_PCG_ shows good agreement (<5% difference). We conclude that the proposed beat to beat fetal heart rate measurement system would be useful for monitoring fetal neurological wellbeing as a better alternative to traditional cardiotocogram based antenatal fetal heart rate monitoring.

## Introduction

Antepartum monitoring of fetal heart rate is an important clinical procedure during pregnancy^1^. The principal aim of antenatal fetal welfare testing is to identify fetuses at risk of intrauterine compromise (e.g. neurological injury) or death, so that these adverse outcomes can be prevented. In recent decades, many techniques for assessment of fetal well-being have been introduced into clinical practice. Despite widespread use of these techniques, there is limited evidence to guide their optimal use or to demonstrate their effectiveness at improving perinatal outcomes. In developed nations, current perinatal mortality rates are approximately 10/1000 births and fetal deaths account for approximately 50% of deaths between 20 weeks of pregnancy and 1 year of age^2^, with congenital malformations and perinatal hypoxia being the principal causes. Even though fetal surveillance (performed more frequently on “high-risk” pregnant groups) may significantly reduce the incidence of fetal deaths, perinatal morbidity and maternal distress in such groups, the majority of stillbirths and malformations now occur in “low-risk” pregnancies (i.e., those with no identified risk factor)^2^. This apparent anomaly emphasizes the urgent need to develop more effective ways of identifying “at-risk” fetuses in “low-risk” groups. In “high-risk” pregnancies, ultrasound-based technologies are the most common diagnostic procedure for identifying fetal compromise, while in “low-risk” groups reduced fetal activity is the only assessment shown to identify fetuses at risk, albeit with poor positive predictive value.

The ultrasound echocardiography is the most frequently used non-invasive method to look at the morphology of the fetal heart with any changes. Although this examination is recommended for high risk pregnant mothers, the instrument is expensive and it requires a well skilled professional for obtaining and evaluating the data.

Fetal Heart Rate (FHR) monitoring^1^ by Doppler based cardiotocography (CTG) in the third trimester is a commonly established method to identify fetal compromises. All pregnancies are usually checked by FHR monitor to identify any abnormality in FHR pattern. The decrease of the FHR (deceleration) indicates an abnormal situation of the pregnancy particularly during uterine contraction^3^. However long term Doppler ultrasound exposure to fetus is not recommended rendering as this technique is not suitable for long term monitoring^4^. Also, the reliability of CTG technique during 24~36 weeks of gestation was reported to be only 60%^5^.

Another novel non-invasive FHR measurement technique is the fetal electrocardiography (FECG) which uses electrodes on the maternal abdominal surface and complex signal processing technique to extract the FECG signals and then estimate the FHR^6,7^. Monica AN24 Monitor can record FHR from fetal ECG data from five electrodes placed on the laboring woman’s abdomen while receiving through skin a preparation with a special abrading solution that removes the stratum corneum of the epidermis in order to optimize signal collection^6^. The main problem is that the signal quality highly depends on the proper electrode placement. Ambient electrical noises generated by other nearby electrical devices, maternal uterine EMG and fetal movement significantly contributed to unstable fetal ECG extraction. Therefore long-term recording can be inconvenient for the mother and may require additional electrodes and their adjustment.

Auscultation is one of the oldest medical tool in history which has also been applied in fetal diagnostics using specially-formed (Pinnard-type) wooden stethoscopes. The modern form of auscultation called phonocardiography, provides the non-invasive electronic recording and computer aided analysis of the acoustic cardiac signals. Because fetal heart activity produces much less acoustic energy and in addition it is surrounded by a highly noisy environment (e.g. acoustic noise produced by the fetal movements, maternal digestive activity, maternal heart activity, movements of the sensor head during recording, external noise originating from the environment), the detection of fetal heart sounds from abdominal phonogram recordings remains a challenging issue. Although several methods have been introduced^8–11^; to the best of our knowledge, currently there are no multichannel fetal phonocardiographic devices available yet.

A cheaper and simpler system would have widespread application in both industrialized and developing countries. In well-resourced countries pregnant women who live far from health facilities could apply the sensors themselves and use a mobile phone to send the data to a remote health worker for analysis and advice. A ‘reassuring’ trace would then avoid the need for expensive and time-consuming travel. In a developing country setting the device could provide interpretation and decision support directly to the health worker at the point of care. This would assist in assessing fetal well-being, detect fetal distress, and help detect mal-presentations and twinning.

A recently published review paper^12^ summarizes the challenges in extracting fetal heart sounds from several abdominal phonography systems in the literature up to date. The major phonogram acquisition problems identified are (1) attenuation of sounds in the transmission path such as amniotic fluid, muscular wall of uterus, layers of fat tissue, bone or cartilage, (2) location of sensor placed on the abdomen in relation to fetal heart or fetal position. The major signal processing challenges identified are (1) low signal to noise ratio makes de-noising very difficult, (2) overlapping frequency bands of fetal and maternal heart sounds and (3) lack of beat by beat validation of fetal heart sounds.

In order to overcome existing challenges in extracting fetal heart sounds reliably and stably, we propose a solution by developing a multi-channel (4-channel in this study) fetal phonocardiogram (fPCG) using multiple sound transducers applied in a simple and consistent pattern across the maternal abdomen, requiring little operator skill. Thus the aim of this study was to compare the beat by beat fetal heart signal acquired by this device with simultaneous measures using a validated fetal electrocardiogram (fECG) device, in healthy pregnancies during the last trimester but before the onset of labour.

## Methods

### Ethical Considerations, Participants and Recruitment

Eligible participants were healthy pregnant women, between 30 and 40 weeks of pregnancy, attending pregnancy outpatient clinic of Tohoku University Hospital in Japan. In order to avoid any interference with needed care for mother or fetus, only those with reassuring CTG traces were recruited. All provided written informed consent to participate, and no identifying information was stored about any participant. The Human Research Ethics Committee of Tohoku University Institutional Review Board (IRB), Sendai, Japan, approved the project. All research was performed in accordance with relevant guidelines/regulations

### Participants

Useful recordings were made from a total of fifteen pregnant women. A summary of their clinical and demographic characteristics is given in Table 1.

**Table 1:** Clinical and demographic characteristics of the study sample (N = 15).

	Age (years)	Gestational Age (weeks)	Body Mass Index
Mean	33.1	39.6	26.0
Range	29–40	36–41	20.0–37.5

### Development of Fetal Phonocardiogram (FPCG) sensors

**Figure 1:**
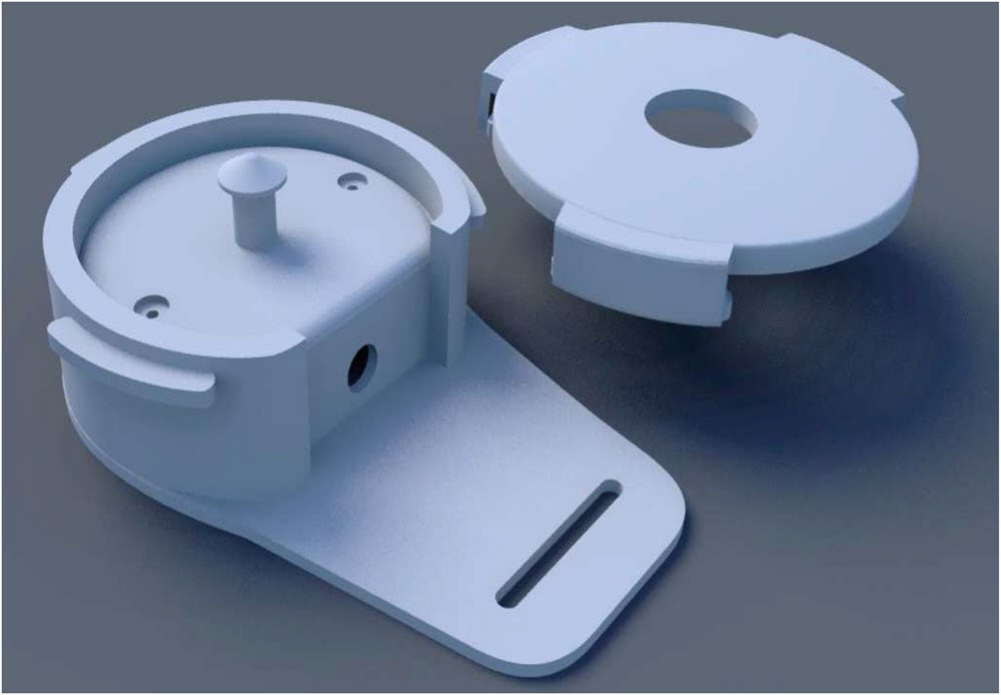
3D model of the phonogram sensor, from the top to the bottom: top cover and container.

**Figure 2:**
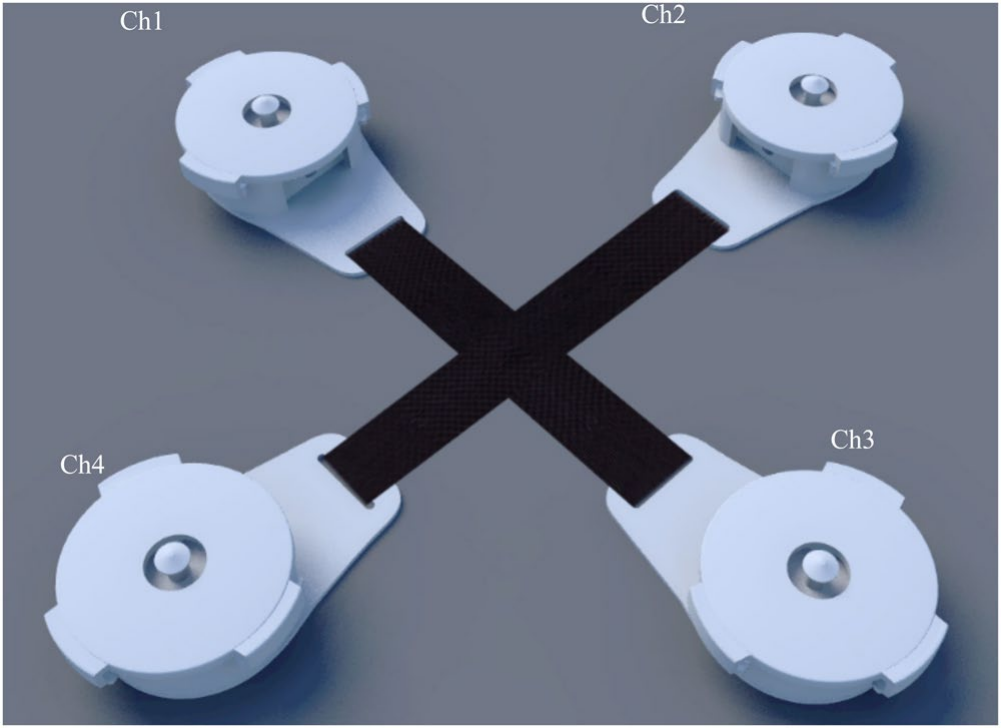
A schematic diagram showing the locations of phonogram sensors. Ch1, Ch2, Ch3 and Ch4 are four phonogram sensors.

In this study, FPCG are recorded using four piezoelectric vibration sensors (cost $1 each) embedded in a high definition 3D printed plastic harnesses as shown in Fig. 1. Each harness holds a ceramic piezo vibration sensor (35 mm diameter) on the maternal abdomen with rubber made cushion to minimize the shear noise. The 3D printed harness is designed with precise parameters that rigidly mount the piezo sensor. The sketch in Fig. 2 shows the setup of the sensors attached with the fabric harness where each sensor picks FPCG signals through a coaxial cable having very high insulating resistance.

### Phonogram Signal recording

**Figure 3:**
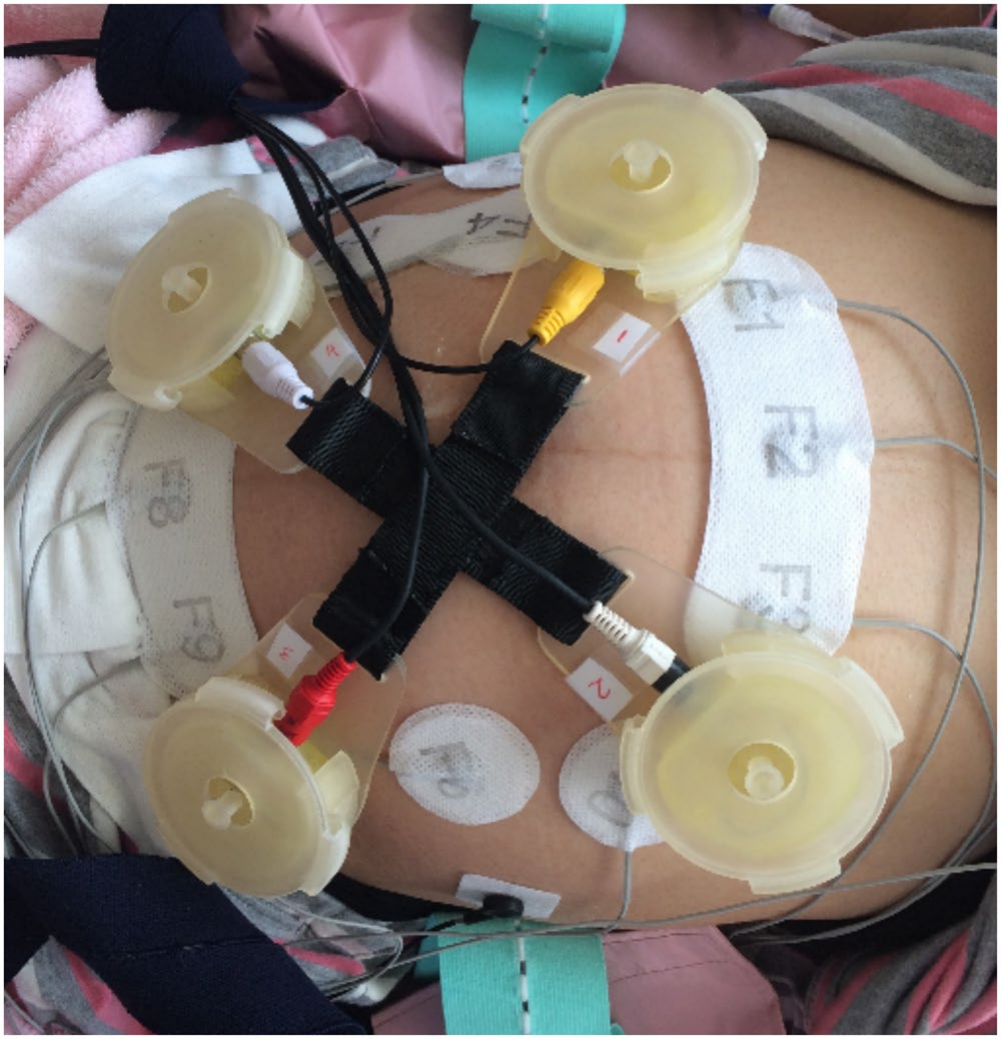
locations of fetal ECG electrodes and phonocardiogram sensors for validation experiment.

**Figure 4:**
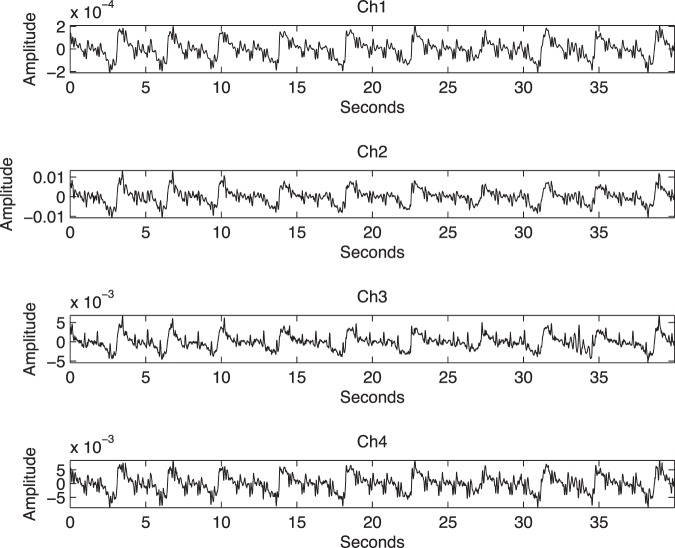
Four-channel unprocessed fetal phonocardiogram signals with 4 FPCG sensors. Ch 1–4 show maternal breathing movement (as low frequency baseline oscillation), and maternal and fetal heart movements (as high frequency oscillations).

The cross point of the harness was placed on the top of belly button of the subject. Four sensors were placed equidistant from that point as shown in Fig. 3. Four channels were amplified and digitized by Powerlab 26T data acquisition system (ADInstruments Inc) and recorded by a laptop computer (fs = 1000 Hz sampling rate, 16 bits resolution) for 10 minutes. The plastic casings and elastic belts proved a simple, efficient and comfortable way to place the sensors in a consistent pattern without the need for previous knowledge of the fetal lie or position. Fetal and maternal heart activity were visible in the unprocessed signals, as was maternal breathing movement shown in Fig. 4.

### Validation of the FPCG sensors

Fetal phonocardiogram signals were captured simultaneously with non-invasive fetal electrocardiogram signals [(IRIS^TM^, Atom Medical Co. Japan] and pulsed Doppler velocimetry for fetal aortic blood flow [Fetal monitor 116, Corometrics, GE Healthcare Inc]. Figure 3 shows how the non-invasive fetal electrocardiogram electrodes were attached (the circular white coloured electrodes in the middle panel), and the four phonogram sensors in their harness.

Agreement between the simultaneous measurements of fetal heart rate was assessed by creating scatter plots and calculating the correlation coefficients and related p-values. Bland-Altman plots were also made, and 95% Limits of Agreement were calculated.

### Fetal electrocardiography

The fetal electrocardiogram was captured non-invasively using twelve electrodes on the surface of the maternal abdomen and thorax and data acquisition system (IRISTM, Atom Medical Co. Japan; PCT/JP2005/023601, PCT/JP2006/316386) with 1000 Hz sampling frequency and 16 bit resolution. The measured signal was analysed essentially as described in our previous study^13^. Twelve electrodes were used for abdominal ECG recordings, ten of which were arranged on the mother’s abdomen, one reference electrode on the back and one electrode was set at the right thoracic position. To separate fetal ECG from the composite abdominal signal, a combination of maternal ECG cancelation and blind source separation with the reference signal (BSSR) was used [13, PCT/JP2005/023601, PCT/JP2006/316386].

### Fetal Phonocardiogram signal processing

**Figure 5:**
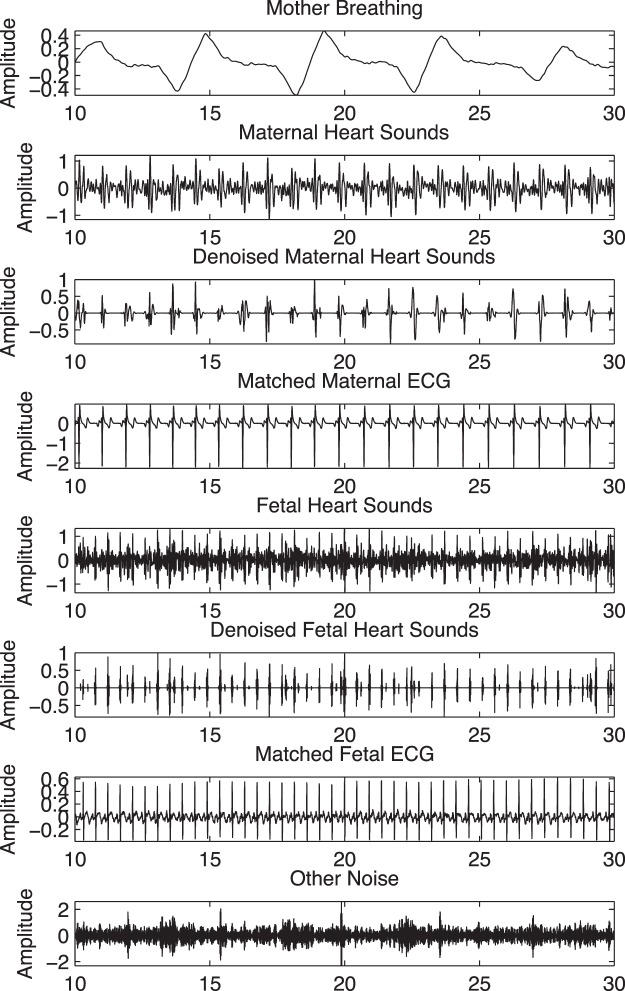
A 20 second example of: extracted fetal heart sound signal (Denoised Fetal Heart Sounds), simultaneously acquired maternal (mECG) and fetal (fECG) ECG signals, extracted maternal heart sound (Denoised Maternal Heart Sounds) and extracted maternal breathing movement (Mother Breathing) signals from 4-channel phonocardiogram sensors, fetal ECG, and maternal ECG.

In this section we briefly visit the decomposition technique used in our previous study^14^. The decomposition technique allowed the raw phonocardiogram signal to be decomposed successfully into separate fetal heart, maternal heart, and maternal respiration sound signals. Figure 5 shows an example of four channel phonogram signals, together with the simultaneous fetal and maternal electrocardiograph signals.

The algorithm described in this section gives an overview of the procedure how we estimated a demixing matrix W^T^ which was able to recover the independent components; such as, the maternal breathing, fetal and maternal heart sounds. A brief description of the Source Separation technique is given as follows.

Let $${{\bf{x}}}^{{\bf{o}}}[{\boldsymbol{n}}]=[\begin{array}{c}Ch1[{\rm{n}}]\\ Ch2[{\rm{n}}]\\ Ch3[{\rm{n}}]\\ Ch4[{\rm{n}}]\end{array}]$$, $${\bf{s}}[{\boldsymbol{n}}]=[\begin{array}{c}fhs[n]\\ mhs[n]\\ mbs[n]\\ noise[n]\end{array}]$$ and A be a well-conditioned square matrix of a maximum size of 4 × 4, as,

1 $${{\bf{x}}}^{{\bf{o}}}[{\boldsymbol{n}}]={\bf{A}}{\bf{s}}[{\boldsymbol{n}}]$$

*fhs[n]* = fetal heart sound; *mhs[n]* = maternal heart sound; *mbs[n]* = maternal breathing sound; *noise[n]* = noise due to movement and fluid motions.

Let **e** be a diagonal control matrix to disable any noisy channel.

2 $${\bf{x}}[{\boldsymbol{n}}]={\bf{e}}{{\bf{x}}}^{{\bf{o}}}[{\boldsymbol{n}}],$$

where the control matrix **e** is defined by

$${\bf{e}}=\,[\begin{array}{cccc}e(1) & 0 & 0 & 0\\ 0 & e(2) & 0 & 0\\ 0 & 0 & e(3) & 0\\ 0 & 0 & 0 & e(3)\,\end{array}]$$

and e(1), e(2), e(3) and e(4) can take logical values of 1 and 0, which are derived from the following matrix.where the correlation coefficient *R*(*i*, *j*) is $\frac{C(i,j)}{\sqrt{C(i,i)C(j,j)}}$, and *C*(*i*, *j*) is the zeroth lag normalized covariance matrix between channels *i* and *j*.

The black squares in the shown matrix indicate mirrored version of the upper triangle cross-correlation coefficients; hence discarded. As shown next to the matrix, we find the *e*(i) values by ORing the correlation coefficient of the current channel with the rest of the channels. We denote the OR operation by || performed on the *R*(*i*, *j*) values after thresholding them, where any *R*(*i*, *j*) value below 0.3 is set to 0 and other values are set to 1.

If we define the observation covariance matrix as **X**[*n*], then for *N* data points,

3 $${\bf{X}}[n]={\bf{x}}[m]{{\bf{x}}}^{{\boldsymbol{T}}}[m+n],$$

where *m* = 0 … *N-n* − 1

Individual item of **X**[*n*] can be calculated using

4 $${X}_{ij}[n]=\sum _{m=0}^{N-n-1}{x}_{i}[m]{x}_{j}[m+n]$$

Equation (4) can also be expressed in terms of mixing matrix **A** as,

5 $${\bf{X}}[n]={\bf{A}}{\bf{s}}[m]{{\bf{s}}}^{T}[m+n]{{\bf{A}}}^{T}={\bf{A}}{\bf{S}}[n]{{\bf{A}}}^{T}$$

where **S**[*n*] is the source covariance (unknown) matrix. If we denote the de-mixing matrix as **W**^T^ where ${{\bf{W}}}^{{\rm{T}}}\,{\bf{A}}={\bf{I}}\,{\rm{or}}\,{{\bf{A}}}^{{\rm{T}}}\,{\bf{W}}={\bf{I}}$, where **I** is the identity matrix, then

6 $${\boldsymbol{s}}[n]={{\bf{W}}}^{{\rm{T}}}{\bf{x}}[n].$$

Equation (5) provides general covariance statistics between shifted versions of the collected sensors readings. Therefore, by using eq. (3) we can define **X**[0], **X**[1] and **X**[*k*] as zero, one and *k*th lag covariance matrices of the sensors’ recordings respectively. These matrices were used to derive the algorithm to recover the sources of a specific set of combination of sensors’ recordings. For examples, for *n* = 0, 1, 2, *k*, we generate eqs 7–10 as,

7 $${\bf{X}}[0]={\bf{A}}{\bf{S}}[0]{{\bf{A}}}^{{\rm{T}}}$$

8 $${\bf{X}}[1]={\bf{A}}{\bf{S}}[1]{{\bf{A}}}^{{\rm{T}}}$$

9 $${\bf{X}}[2]={\bf{A}}{\bf{S}}[2]{{\bf{A}}}^{{\rm{T}}}$$

10 $${\bf{X}}[k]={\bf{A}}{\bf{S}}[k]{{\bf{A}}}^{{\rm{T}}}$$

In this study, we assumed that the sources are non-white and decorrelated, therefore, we used the same mixing matrix **A**^T^. If the *k*th sample is the discrete time lag over a very short period, the sum of the *k*th lag covariance matrices up to *k* is given by the following equation.

11 $$\sum _{n=1}^{k}{\bf{X}}[n]={\bf{A}}(\sum _{n=1}^{k}{\bf{S}}[n]){{\bf{A}}}^{{\rm{T}}}$$

Multiplying each right side of eq. (11) by a desired demixing matrix W results in the following equation.

12 $$(\sum _{n=1}^{k}{\bf{X}}[n]){\bf{W}}={\bf{A}}(\sum _{n=1}^{k}{\bf{S}}[n]),$$

and the original mixing matrix **A** can be rewritten as,

13 $$(\sum _{n=1}^{k}{\bf{X}}[n]){\bf{W}}{(\sum _{n=1}^{k}{\bf{S}}[n])}^{-1}={\bf{A}},$$

To better approximate the demixing matrix **W**^T^, we aim to find the minimum sum to *k*th lag covariance matrix which constructs an eigenvalue decomposition sets. By substituting **A** in eq. (13) in the zero lag covariance form derived in eq. (7), we get the following equation.

14 $${\bf{X}}[0]{\bf{W}}=(\sum _{n=1}^{k}{\bf{X}}[n]){\bf{W}}{(\sum _{n=1}^{k}{\bf{S}}[n])}^{-1}{\bf{S}}[0]$$

Equation (14) can be further simplified as,

15 $${\bf{X}}[0]{\bf{W}}=(\sum _{n=1}^{k}{\bf{X}}[n]){\bf{W}}{\boldsymbol{\Lambda }}$$

where ${\boldsymbol{\Lambda }}={(\sum _{n=1}^{k}{\bf{S}}[n])}^{-1}{\bf{S}}[0]$ is a diagnonal matrix and all **S**[0], **S**[1] … **S**[*k*] have the same diagonalization property over a shor time period.

The expression in eq. (15) is in the form that produces a diagonal matrix **Λ** of generalized eigenvalues and a full matrix **W** whose columns are the corresponding eigenvectors that maximize the decorrelation between the collected signals. The idea here is that the full matrix **W** is nothing but the eigenvectors matrix constructed not only from the covariance matrix **X**[0], but also from the sum of the minimum *k*th lag cross covariance matrix ${\sum }_{n=1}^{k}{\bf{X}}[n]$. Because the sources are non-white, this method is described as using second order statistics in the form of covariance matrices for different time lags.

**Steps through application:**

1. Construct the input matrix: ${\bf{x}}[{\boldsymbol{n}}]={\bf{e}}{{\bf{x}}}^{{\bf{o}}}[{\boldsymbol{n}}]$.
2. Find **X**[0].
3. Find ${\sum }_{n=1}^{k}{\bf{X}}[n]$, up to predefined *k*th-lag sample.
4. Construct the eigenvalue decomposition problem: $\,[{\bf{W}},{\boldsymbol{\Lambda }}]={\rm{eig}}({\bf{X}}[0],{\sum }_{n=1}^{k}{\bf{X}}[n])$.
5. Find the sources: ${\boldsymbol{s}}[n]={{\bf{W}}}^{{\rm{T}}}{\bf{x}}[n]$.

## Results

### Beat by beat matching of fetal and maternal heart heats

**Figure 6:**
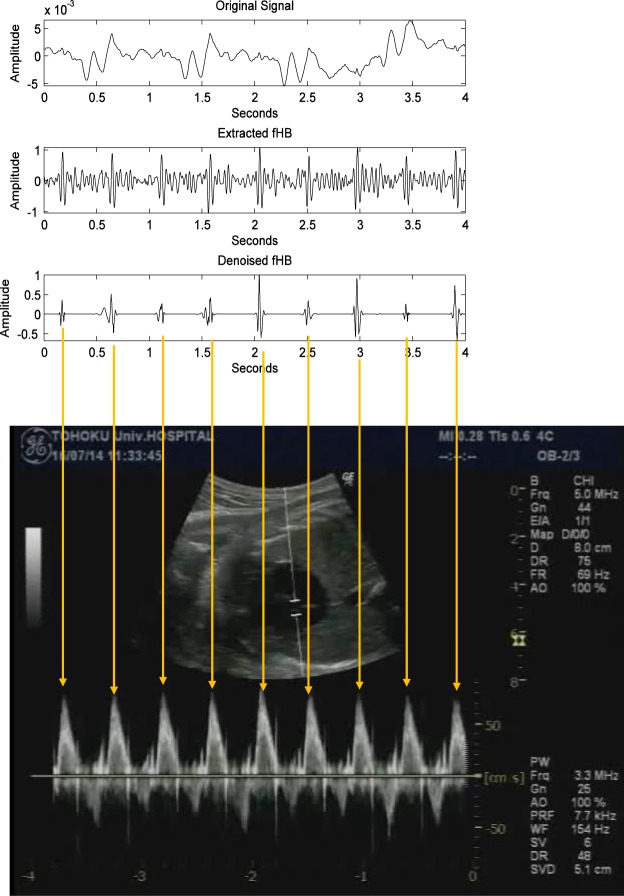
Beat by beat validation with pulsed Doppler M-mode images.

The simultaneous recordings of fetal and maternal ECG signals and extracted fetal and maternal phonocardiogram signals are shown in Fig. 5. To further confirm the fetal heart beats, simultaneously recorded M-mode images of fetal aortic blood flow were used as shown in Fig. 6.

### Denoising fetal and maternal heart sounds

The extracted signals were further denoised using a wavelet transform-based stationary-non-stationary filter (WTST-NST)^15^. The WTST-NST utilizes a multi-resolution wavelet decomposition with threshold values at each level to separate non-stationary signal components (noise) from stationary signal components (heart sounds). We used db1 family with threshold level at 3 standard deviation of the wavelet coefficients. The algorithm keeps decomposing into levels until no further details can be extracted leaving the stationary parts alone (fetal heart sounds or maternal heart sounds), while the non-stationary part as noise. Denoised maternal and fetal heart sounds as shown in the 3^rd^ and 6^th^ panels of Fig. 5 clearly removed the baseline noises. Peaks were detected from filtered fetal and maternal heart sound signals.

### Comparison of Fetal Phonocardiogram and Electrocardiogram

**Figure 7:**
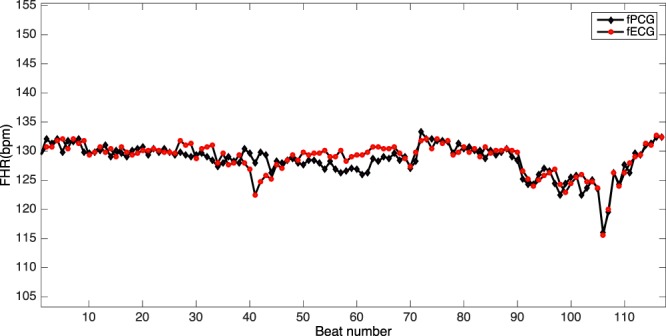
From a 38-week pregnancy: the instantaneous fetal heart rate estimated from simultaneous fetal ECG signals (black dotted line) and phonocardiogram signals (red line).

**Figure 8:**
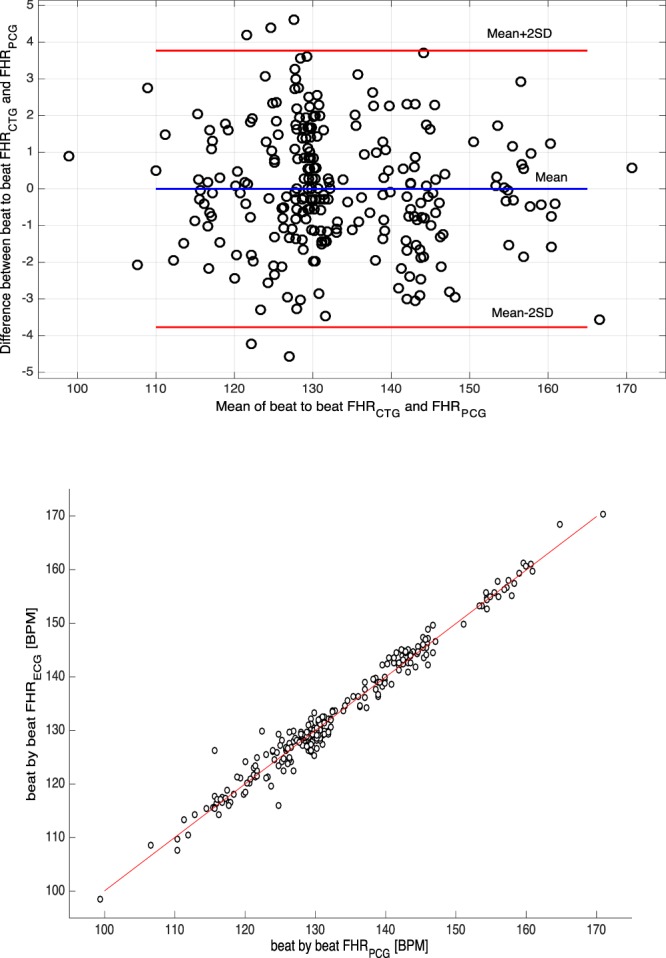
(Upper panel) Bland Altman plot of the relationship of the difference between mean fetal heart rate from ECG (FHR_ECG_) and from phonocardiogram signals (FHR_PCG_) versus their average values from 270 heart beats collected from 15 fetuses (33–40 weeks). Mean bias (—), +2 SD and −2SD lines are shown. SD = standard deviation. (Lower panel) Correlation between FHR_ECG_ and FHR_PCG_ (r = 0.96; p < 0.01).

Figure 7 shows that the fetal heart rate calculated from the Phonocardiogram was very similar to fetal heart rate calculated from the fetal ECG. The mean instantaneous fetal heart rate (i.e. calculated from the beat-to-beat interval) for a single participant over 120 heart beats, illustrates the very good correlation between the two. Figure 8 (upper panel) is a Bland-Altman plot of fetal heart rate by fECG vs Phonocardiogram for the 15 participants: the 95% Limits of Agreement were from −11.3 to 8.6 beats/min and the mean difference was −1.3 beats/min (95% CI −3.20–0.53 beats/min). Figure 8 (lower panel) plots the strong correlation between fetal heart rate by fECG and Phonocardiogram for the same participants: r = 0.96, with p < 0.01. Table 2 shows that RMSSD of FHR extracted from fPCG signals is significantly different from fECG based method.

**Table 2:** Comparison of fetal heart rate (FHR) and heart rate variability (from 1 minute heart rate time series) extracted from fetal ECG and fetal Phonocardiogram (FPCG) recordings.

Feature	Fetal ECG (N = 15)	Fetal PCG (N = 15)	p value
Mean FHR (bpm)	146 ± 8	145 ± 12	0.34
SDNN FHR (bpm)	6.27 ± 2.9	6.85 ± 2.81	0.13
RMSSD FHR (bpm)	2.69 ± 1.29	3.89 ± 2.74	0.01*

*Means significance (p < 0.01) between HRV and PRV during normal breathing events.

## Discussion

Changes in beat-to-beat fetal heart rate on short term and long term are associated with activities of parasympathetic and sympathetic branches of autonomic nervous system^16,17^. Beat to beat variations of fetal heart rates were reported to be lost in stillbirth and severe birth academia^18,19^.

This study provides initial evidence to support a multi-channel phonogram as a feasible way to capture fetal heart sounds on beat by beat from the maternal abdominal surface. The multi-channel approach means that at least one sound transducer is always close enough to the fetal heart to provide suitable data, regardless of the fetal position. Maternal heart sounds and breathing movements can also be detected and monitored at the same time. Data from fifteen participants show that the fetal heart rates, both instantaneously and over several minutes, correlate well with rates determined by simultaneous fetal electrocardiography.

Standardized placement of the transducers was achieved through the use of a simple elastic harness, so the sensors could be placed by an operator with relatively little skill. It is likely that any pregnant women should be able to correctly apply the sensors and harness by themselves at home, and in future the data could be transmitted by mobile phone to a distant midwife or obstetrician for interpretation. This could lead to cost and time savings where the results are reassuring. The participants found the harness and sensors caused no discomfort, and the sensors are completely non-invasive, suggesting that this approach should be useful for longer periods of continuous monitoring.

Phonogram signal processing has been a challenging issue in this study. Recordings of Abdominal phonogram signals are affected by acoustic damping of the amniotic fluid, maternal abdominal tissues and fats, external environmental sound noise, physiological activity from the mother (i.e. bowel activity, blood flow, heart and respiratory sounds) and shear noises between maternal abdominal wall and sensor surface area. Channel to channel inter-correlations were needed to exclude the signals from dysfunctional sensors and eigenvector matrix which was prepared from *k*th lag (k = 6) covariance matrix was necessary to separate fetal and maternal heat sounds. Fetal heart is smaller in size, less powerful and beats at a faster rate than maternal heart. Hence, frequency range of fetal heart sounds is higher and wider than the same of maternal ones. Because eigenvector matrix was generated from phonogram signals, external inputs from other sources were not necessary in this study. Thirty iterations for finding the best demixing matrix was performed on different panels of each subject to converge to the source separation technique in the present study. The multilevel wavelet decomposition with dynamic threshold values at each level to separate non-stationary signal components (noise) from stationary signal components (heart sounds) was found to be effective in denoising extracted noisy fetal and maternal heart sounds. Because the proposed technique involves decomposition of the sources using extracted eigenvectors, it could potentially be applied for fast in real-time processing of fetal and maternal heart sounds in mobile platform.

This was a preliminary study: we did not test the device for the detection of fetal heart rate abnormalities, nor did we test it during labour or outside the third trimester. Further studies should include larger numbers of participants with a wider range of body habitus and a wider range of gestational ages. Studies will be required in abnormal pregnancies and during labour, to establish the usefulness of this novel multi-channel sound-transducer-based approach to fetal monitoring. It has not been tested yet to compare with STAN intrapartum method. However, the results are encouraging. This novel approach is likely to provide a low-cost alternative to traditional cardiotocography, with the advantage of lower operator skill requirements and a fully non-invasive technique.
